# Protective role of maize purple plant pigment against oxidative stress in fluorosis rat brain

**DOI:** 10.1515/tnsci-2020-0055

**Published:** 2020-04-21

**Authors:** Boyan Li, Keyana Nozzari Varkani, Lu Sun, Bo Zhou, Xiaohong Wang, Lianying Guo, Han Zhang, Zhuo Zhang

**Affiliations:** Department of Nutrition and Food Hygiene, School of Public Health, Shenyang Medical College, Shenyang, China; Department of Clinical Medicine, School of International Education, Shenyang Medical College, Shenyang, China;; Department of Radiological Health, Center for Disease Prevention and Control of Liaoning Province, Shenyang, China

**Keywords:** fluoride, oxidative stress, anthocyanins, rats

## Abstract

In fluorosis-endemic areas, exposure to high levels of fluoride causes neurotoxicity such as lowered intelligence and cognitive impairment. Oxidative damage is critical to pathophysiologic processes of fluoride intoxication, and neurotoxicity of fluoride may be associated with oxidative stress. In previous studies, maize purple plant pigment (MPPP), which was rich in anthocyanins, showed a strong scavenging activity *in vitro* and *in vivo*. The present study aimed to determine whether treatment with MPPP can alleviate fluoride-induced oxidative damage in rat brain. After 3 months of experiment, brain tissues were assayed for oxidative stress variables, histological and Western blotting examinations. Our results showed that MPPP reduced the elevated malondialdehyde levels, increased superoxide dismutase activity, and further attenuated histopathological alterations and mitigated neuronal apoptosis. Importantly, MPPP also reversed changes in Bax and Bcl-2. Therefore, it was speculated that MPPP protects brain tissue from fluoride toxicity through its antioxidant capacity.

## 1 Introduction

Given the widespread presence of fluorine in the natural environment, individuals are exposed to fluoride via food intake, inhalation, and dermal contact. Drinking water represents the largest exposure source. In particular, in highly fluoridated regions and in some developed areas that fluoridate the public water supply to reduce dental caries, fluoride may result in a health hazard [1,2].

Fluoride is required for normal growth and development of teeth and bones but can lead to fluorosis if taken excessively. Specifically, fluorosis can adversely affect the skeleton and teeth, and may induce structural and functional changes in soft tissues including brain tissue [3]. Epidemiological data show that chronic exposure to high fluoride in water is closely associated with a lower intelligence quotient in children [4,5,6]. In fluorosis-endemic areas, a certain high dose of fluoride intake is a potential risk factor for cognitive impairment in elderly people [7]. Moreover, structural changes in nerve cells and brain functions in experimental animals subjected to chronic fluorosis have been described such as nuclear shrinkage, mitochondrial swelling, neurodegeneration, and deterioration of learning and memory [8,9,10]. These findings suggest a direct link between excessive exposure to fluoride and brain function impairment, but little is known about mechanisms underlying these phenomena.

Oxidative stress-induced neurotoxicity is considered a mechanism of brain impairment caused by fluorosis. Once fluoride has formed lipid-soluble complexes in the blood, it can cross the blood–brain barrier, penetrate brain cells, and accumulate in brain tissue, causing detrimental neurological effects [11]. Reactive oxygen species (ROS) and free radicals can be generated when the fluoride content is high in the brain and cause oxidative damage and cell apoptosis in neurons [12], which may be controlled by apoptosis-related genes [13,14,15]. The literature suggests that increased ROS and lipid peroxidation (LPO) and decreased antioxidant enzyme activity occur in the brains of fluoride-intoxicated rats and that histopathological changes can be observed, especially swelling of mitochondria and endoplasmic reticulum dilation in neurons [9,16]. Also, some studies confirm that specific antioxidants may protect against this damage [17].

Anthocyanins, the largest group of water-soluble pigments responsible for fruit and vegetable color, are flavonoids reputed to have biological antioxidant activity due to their capacity as hydrogen donors [18]. They can also stabilize and delocalize unpaired electrons, and their ability to chelate transition metal ions may be useful [19]. Anthocyanin-rich maize purple plant pigment (MPPP) extracted from maize purple plant has been said to have antioxidant traits [3,20], but few reports of MPPP in fluoride-treated rat brains exist. Thus, we studied MPPP and any potential neuroprotective effects against fluoride toxicity.

## 2 Materials and methods

### 2.1 Chemicals and reagents

Sodium fluoride (NaF, molecular weight 41.99) was procured from Sigma Chemical (St. Louis, MO, USA). Anti-Bax and anti-Bcl-2 antibodies were obtained from Santa Cruz Biotechnology (Santa Cruz, CA, USA). All other laboratory reagents used were of analytical grade and obtained from Sigma, Invitrogen (Carlsbad, CA, USA) and Sangon Biotech Co., Ltd (Shanghai, China). MPPP extracted and separated from maize purple plant was produced by Liaoning Dongya Seeds Co., Ltd (Shenyang, China). In our previous study, we confirmed that MPPP mainly contains 45.96% cyanidin-3-glucoside, 12.99% 3′,4′-dihydroxy anthocyanin-3-glucoside, and 26.16% four other kinds of anthocyanins [21]. MPPP mixed with the standard rodent diet was obtained from Shenyang Qianmin Animal Feeds Factory (Shenyang, China).

### 2.2 Animals and treatment

Eighty healthy weanling Wistar rats (50% male) were acclimated for 1 week before experiments and fed a common basal pellet diet and water ad libitum. The rats were randomized into four groups (*N* = 20/group) by body weight stratification. Group I (controls) received tap water and a common basal pellet diet for 12 weeks. Group II (fluoride-treated rats) received 100 ppm fluoride ion (F^−^) in their drinking water and a common basal pellet diet for 12 weeks. Group III (experimental rats co-treated with fluoride and MPPP) received 100 ppm F^−^ in their drinking water and pellet diet mixed with 5 g/kg MPPP for 12 weeks. Group IV (experimental rats co-treated with fluoride and MPPP) received 100 ppm F^−^ in drinking water and a pellet diet mixed with 10 g/kg MPPP for 12 weeks. During treatment, daily water consumption, animal feed consumption and weight gain were recorded periodically. Fluoride and MPPP intake was calculated according to weekly average water and animal feed consumption. All rats were kept in ventilated cages at 23–27°C, with 55–60% humidity and 12/12 h light/dark cycles. After 12 weeks, the treatments were ended, and the rats were killed under light ether anesthesia.

All brain tissues were dissected carefully and blotted free of blood, and their fresh weight was recorded. Brain somatic indices were calculated as g/100 g weight by the following formula: fresh weight of the brain/weight of the body × 100. Ten brain tissue samples were selected randomly from each group for the F^−^ assay and Western blotting; two other brain tissue samples from each group were fixed in 2.5% glutaraldehyde for ultrastructural examination. The remaining brain tissues were homogenized in chilled potassium chloride and centrifuged at 3,000 × *g* for 10 min at 4°C. The supernatant was used for biochemical analysis.


**Ethical approval:** This research related to animal use complied with the Guidelines for the Care and Use of Laboratory Animals of the China National Institute of Health. The experimental protocols were approved by the Ethics Committee for Animal Experiments of Shenyang Medical College (permit number S-2013-006). All surgeries were performed under light ether anesthesia, and all efforts were made to minimize suffering.

### 2.3 Determination of fluoride

Following published methods [22], brain tissue samples (50 mg) digested with lipase and protease were dissolved in an acid mixture (nitric acid and silver nitrate) in a closed compartment, which was overlaid with saturated sodium hydroxide. After neutralization for 24 h, fluorine reagent was added into the mixture, and F^−^ in brains was calculated from a standard curve. Data were expressed as μg F^−^/kg brain tissue.

### 2.4 Ultrastructure of brain

Brain tissues were fixed with 2.5% glutaraldehyde for 2 h and 1% osmium tetroxide for another 2 h. Subsequently, samples were dehydrated through a graded ethanol series and embedded in Spurr’s resin. Ultrathin sections were cut and stained with uranyl acetate and lead citrate and then observed and photographed using a Hitachi H-7650 (Hitachi Ltd, Tokyo, Japan) transmission electron microscope.

### 2.5 Brain tissue oxidative stress markers

LPO was assessed via malondialdehyde (MDA) in rat brains. MDA, glutathione (GSH), glutathione peroxidase (GSH-Px), and superoxide dismutase (SOD) activity in brain tissue was assayed using commercial kits (Jiancheng Bioengineering Institute, Nanjing, China). Total proteins were measured using the Bradford assay to normalize MDA, GSH, GSH-Px, and SOD [23]. Data are expressed as nmol/mg protein for MDA, U/mg protein for SOD and GSH-Px, and mg/g protein for GSH in brain tissues.

### 2.6 Bax and Bcl-2 expression in rat brains

Frozen brain tissue samples were placed in ice-cold lysis buffer, homogenized at low temperature, and then centrifuged at 4°C at 12,000 × *g* for 25 min. Protein in the supernatant was quantified using the protein assay kit. Lysates with equal amounts of protein were separated on 10% SDS-PAGE and electrotransferred to a polyvinylidene difluoride membrane (Millipore, Bedford, MA, USA), which was blocked with 5% non-fat dried milk in Tris-buffered saline with Tween 20 for 1.5 h at room temperature. Thereafter, membranes were incubated with primary antibodies against Bcl-2 and Bax (1:1,000) overnight at 4°C. Next, horseradish peroxidase-conjugated secondary antibody (1:6,000) was applied for 1 h at room temperature. After rinsing with buffer, protein bands were visualized with an enhanced chemiluminescence reagent and analyzed by Gel-Pro Analyzer software. β-Actin was used as a protein-loading control.

### 2.7 Statistical analysis

All data were analyzed using SPSS v17.0 software (SPSS Inc., Chicago, IL, USA) and analyzed by one-way analysis of variance followed by Dunnett’s test to compare mean values between different treatment groups. Experimental results are expressed as mean ± standard error of mean (SEM), and *p* < 0.05 was considered to be statistically significant.

## 3 Results

**Figure 1:**
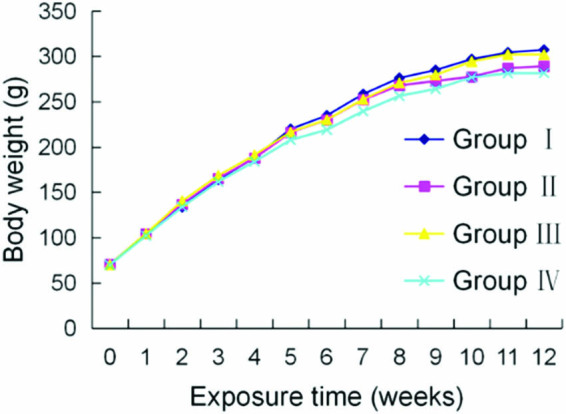
Effects of fluoride and MPPP on rat weight. Data are mean values of 20 replicates.

No clinical signs of toxicity were observed in any group of rats throughout the dosing period of 12 weeks. Body weight in fluoride-treated animals decreased slightly as shown in Figure 1. Brain fluoride in fluoride-treated groups increased significantly compared with controls, and MPPP at both doses reduced this fluoride but not significantly. The brain somatic index showed no significant differences among all of the groups (Table 1).

**Table 1:** Levels of fluoride in brain and brain somatic index of rats (*x* ± SEM)

Groups	Levels of fluoride (μg/kg)	Brain somatic index (g/100 g)
Group I	210.28 ± 53.25	0.64 ± 0.04
Group II	800.21 ± 79.82*	0.67 ± 0.03
Group III	698.65 ± 111.32*	0.66 ± 0.03
Group IV	600.16 ± 37.40*	0.69 ± 0.03

Note: *p < 0.05 compared with the control group (group I). *N* = 10 in each group for levels of fluoride; *N* = 20 in each group for measurement of the brain somatic index.

### 3.1 Ultrastructural observation of brain

**Figure 2:**
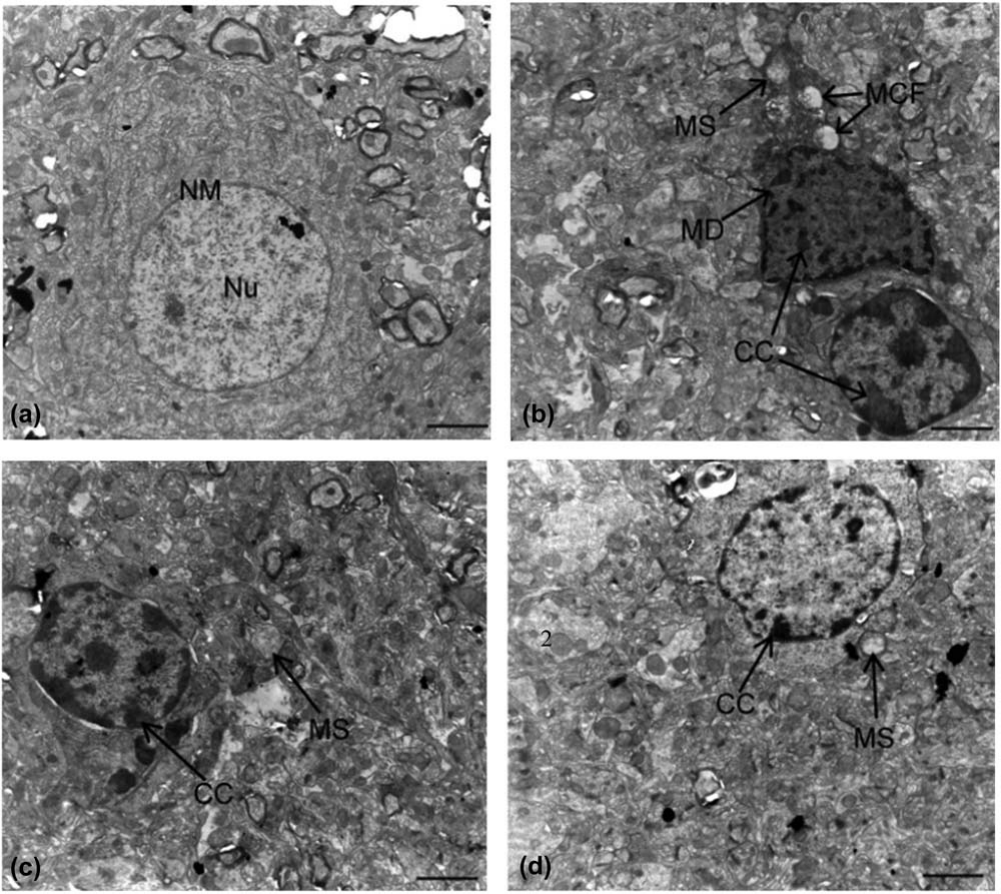
Ultrastructure of nerve cells in rats. (a) Controls; (b) rats fed 100 mg/L fluoride; (c) rats fed 100 mg/L fluoride plus 5 g/kg MPPP; and (d) rats fed 100 mg/L fluoride plus 10 g/kg MPPP. Nu, nucleolus; NM, nuclear membrane; MD, membrane dissolution; MS, mitochondrial swelling; CC, chromatin condensation; and MCF, mitochondrial crest fracture. Bar = 2 µm.

Ultrastructural analysis of the experimental rat brains is shown in Figure 2. For controls (Figure 2a), one oval nucleus with visible, clear nucleoli and double nuclear membranes, abundant mitochondria, and endoplasmic reticulum were found in neurons. In fluoride-treated rats (Figure 2b), nerves were deformed, lacked a nuclear membrane, and had chromatin condensation, swollen mitochondria, and broken cristae, and evidence of apoptosis was present. In rats treated with fluoride and MPPP, brain cells had swollen mitochondria but fewer abnormal mitochondria compared to group II, and pathological nuclear changes were reduced (Figure 2c and d).

### 3.2 Oxidation in rat brains

**Figure 3:**
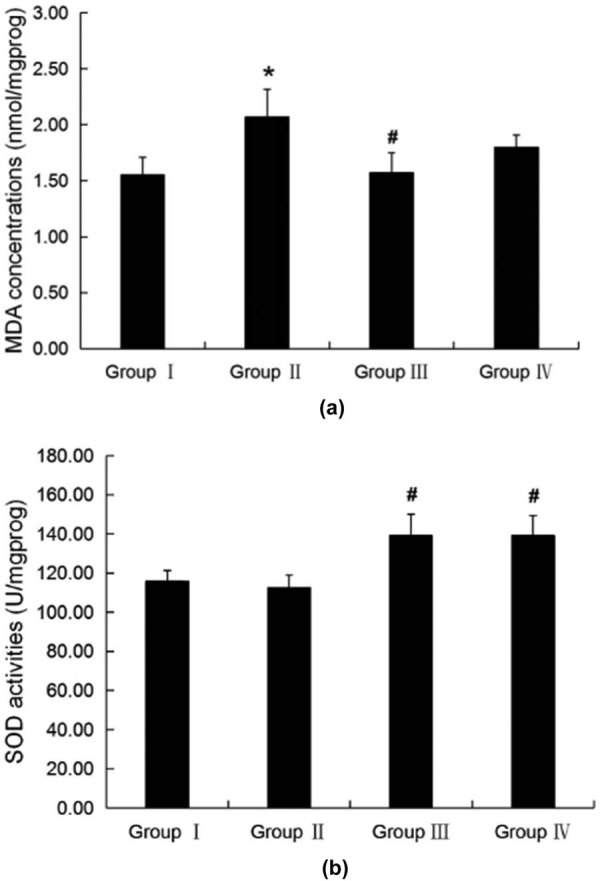
LPO and antioxidant status in rat brains. (a) LPO production (MDA) and (b) SOD. *p < 0.05 compared with group I; #*p* < 0.05 compared with group II.

MDA, GSH, SOD, and GSH-Px activities were assessed, and the MDA level was significantly greater for the fluoride-treated rats than for the controls. MPPP (5 g/kg) reduced the elevated MDA after fluoride, and SOD increased (groups III and IV) compared with rats treated with fluoride alone (*p* < 0.05), as shown in Figure 3. GSH-Px activity and GSH were only slightly increased in groups III and IV compared with the fluoride-treated rats group (data not shown).

### 3.3 Bax and Bcl-2 expression in rat brains

**Figure 4:**
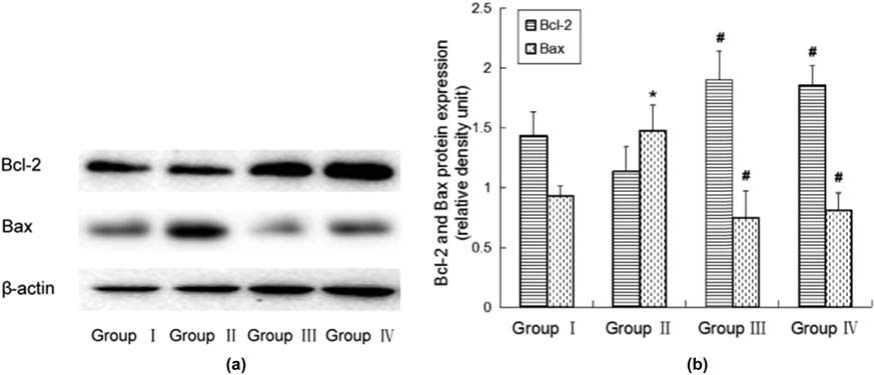
Bcl-2 and Bax protein in rat brains. (a) Western blot and (b) the relative densitometry of the bands. Relative protein expression was normalized to β-actin. Bars are mean ± SEM. *p < 0.05 compared with group I, #*p* < 0.05 compared with group II.

Bax and Bcl-2 protein expression in brains as measured by Western blotting (Figure 4) showed that Bax increased in fluoride-treated rats compared with controls and also decreased at both doses of MPPP. Bcl-2 protein expression in rat brains after MPPP treatment was significantly elevated compared with fluoride-treated rats.

## 4 Discussion

Because soluble fluoride is absorbed easily from the gastrointestinal tract, high concentrations of fluorine from drinking water may accumulate, but only the water-soluble fluoride ion is relevant to human health [2]. Neurotoxic severity chiefly depends upon the content of fluoride in drinking water when exceeding the WHO-recommended value of 1.50 ppm [24,25].

Chronic ingestion of high concentrations of fluoride can induce excessive production of oxygen free radicals with subsequent LPO in soft tissues [26]. Oxidative imbalance due to increased free radicals can cause oxidative damage in fluoride-intoxicated animals [27], especially the brain, which has a high content of polyunsaturated fatty acids. Children from areas of endemic fluorosis have lower intelligence and elevated oxidative stress status, as measured by increased MDA [28]. MDA, an aldehydic product of membrane LPO, is often used as a marker of oxidative stress in tissues. We noted that MDA in brain tissues in fluoride-treated rats was significantly higher than in controls, as noted in previous studies [29,30]. Excessive fluoride intake can promote oxidative stress and disturb the antioxidant defense system in brains of fluoride-intoxicated rats, so it has been suggested that antioxidants (vitamins, resveratrol, and anthocyanins) and antioxidant-rich foods (such as rhodiola) may be useful for reducing such damage [17,31,32,33].

Anthocyanins, water-soluble natural plant pigments, have been reported to have antioxidant effects [18]. Furthermore, one study suggests that consumption of anthocyanins can reduce free radicals in the body [34], likely via scavenging superoxide anion radicals [35], inhibiting LPO, and interfering with hydroxyl radical-generating systems [36]. Anthocyanin-rich MPPP appeared to have antioxidant properties as we observed that MPPP (5 g/kg feed) significantly reduced MDA in brains of fluoride-intoxicated rats, and SOD activity in rat brains after MPPP treatment (5 and 10 g/kg feed) was significantly elevated. Thus, MPPP may capture free radicals and enhance endogenous antioxidant activity.

Chronic fluorosis can cause brain structural and functional changes via oxidative stress after fluoride exposure [8,9,10], and we noted histological changes in the brains of fluoride-treated rats characterized by cell nucleus deformation, chromatin condensation, and swollen mitochondria and typical morphological manifestations of apoptosis. Similar observations were made by others who reported cytomorphosis, intranuclear heterochromatin margination condensation, and shrinkage of the nucleus in the brains of fluoride-intoxicated mice [37]. MPPP may have alleviated the harmful effects of fluoride by increasing SOD activity and reducing MDA. Neuronal apoptosis, which has been reported in the presence of relatively high fluoride, was allegedly due to increased oxidative stress [13]. Apoptosis can be coordinately controlled by gene expression, so we measured the apoptosis-promoting Bax protein and the inhibitory Bcl-2 protein, which are abundant in mitochondria, the nuclear membrane, and endoplasmic reticulum. Others reported that Bax protein expression is significantly upregulated in the brains of fluoride-treated rats, and a negative correlation was observed between fluoride concentrations in water and expression of Bcl-2 [37]. We report that Bax protein expression was significantly increased and expression of Bcl-2 protein decreased in the brains of fluoride-treated rats. Also, MPPP prevented the decrease in Bcl-2 and the increase in Bax expression after fluoride treatment, suggesting that MPPP alleviates apoptosis-mediated impairments.

MPPP did not modify fluoride ions in brain tissues; thus, reducing fluoride exposure is not a mechanism underlying its purported ability to reduce fluoride toxicity.

## 5 Conclusions

Oxidative stress plays a role in fluoride-induced toxicity and provokes pathological changes and neuronal apoptosis in rat brains. Anthocyanin-rich MPPP may restore brain health via its antioxidant properties. However, further research is required to understand how MPPP may be neuroprotective.
